# A Novel Framework for Image Matching and Stitching for Moving Car Inspection under Illumination Challenges

**DOI:** 10.3390/s24041083

**Published:** 2024-02-07

**Authors:** Andreas El Saer, Lazaros Grammatikopoulos, Giorgos Sfikas, George Karras, Elli Petsa

**Affiliations:** 1 Department of Surveying and Geoinformatics Engineering, University of West Attica, 12243 Athens, Greece; elsaer@uniwa.gr (A.E.S.); gsfikas@uniwa.gr (G.S.); petsa@uniwa.gr (E.P.); 2 School of Rural, Surveying and Geoinformatics Engineering, National Technical University of Athens, 15780 Athens, Greece; gkarras@central.ntua.gr

**Keywords:** image matching, feature extraction, structure from motion, deep learning, car inspection, moving objects, glossy surfaces, outlier removal, image pose estimation

## Abstract

Vehicle exterior inspection is a critical operation for identifying defects and ensuring the overall safety and integrity of vehicles. Visual-based inspection of moving objects, such as vehicles within dynamic environments abounding with reflections, presents significant challenges, especially when time and accuracy are of paramount importance. Conventional exterior inspections of vehicles require substantial labor, which is both costly and prone to errors. Recent advancements in deep learning have reduced labor work by enabling the use of segmentation algorithms for defect detection and description based on simple RGB camera acquisitions. Nonetheless, these processes struggle with issues of image orientation leading to difficulties in accurately differentiating between detected defects. This results in numerous false positives and additional labor effort. Estimating image poses enables precise localization of vehicle damages within a unified 3D reference system, following initial detections in the 2D imagery. A primary challenge in this field is the extraction of distinctive features and the establishment of accurate correspondences between them, a task that typical image matching techniques struggle to address for highly reflective moving objects. In this study, we introduce an innovative end-to-end pipeline tailored for efficient image matching and stitching, specifically addressing the challenges posed by moving objects in static uncalibrated camera setups. Extracting features from moving objects with strong reflections presents significant difficulties, beyond the capabilities of current image matching algorithms. To tackle this, we introduce a novel filtering scheme that can be applied to every image matching process, provided that the input features are sufficient. A critical aspect of this module involves the exclusion of points located in the background, effectively distinguishing them from points that pertain to the vehicle itself. This is essential for accurate feature extraction and subsequent analysis. Finally, we generate a high-quality image mosaic by employing a series of sequential stereo-rectified pairs.

## 1. Introduction

Exterior vehicle inspection and defect monitoring have evolved significantly over the years, driven by advancements in computer vision and deep learning with an increasing focus on road safety and efficiency [1,2,3,4,5,6]. These technologies transform the field of vehicle exterior inspection by automating defect detection, classification and segmentation processes. This not only accelerates the inspection process but also enhances the accuracy of damage and defect assessment, leading to more reliable and cost-effective solutions. Recent progress in deep learning can reduce manual labor by enabling the use of segmentation algorithms for defect detection and description utilizing image sequences.

Additionally, deep learning can allow intelligent fault diagnosis and predictive maintenance. This involves identifying, isolating and correcting faults, for instance, by transferring knowledge between a source well-studied model to a target model through transfer learning [7,8]. Moreover, similar approaches are capable of running at the edge [9] for increased efficiency and cost-effectiveness.

Nonetheless, these processes struggle with image orientation, leading to difficulties in accurately differentiating between detected defects. This results in numerous false positives (re-detections) and additional labor and effort. To enable precise defect localization and distinction, accurate image poses ensuring a clear understanding of the overlap and relative positions of detected defections or damages should be estimated. This orientation is vital to minimize false positives and streamline the inspection process.

Image matching is a fundamental process in computer vision, essential for identifying correspondences between two or multiple images. This process, which encompasses feature detection, description and matching, has traditionally been addressed through tools such as SIFT [10], SURF [11] and ORB [12], depending on the particular use case. While these handcrafted techniques have been widely utilized, the rise of deep learning has paved the way for more sophisticated approaches, providing solutions resilient to image matching challenges.

Innovative methods such as SuperPoint [13], LoFTR [14] and Roma [15], which have recently emerged, demonstrate improved feature detection and matching capabilities that surpass previous methods, particularly in challenging scenarios like poor texture or repetitive patterns. Despite these advances, image matching in dynamic scenes with moving vehicles and reflections remains a formidable challenge. Such conditions in vehicle exterior monitoring frequently result in redundant detections of defects, thereby increasing the manual workload for verification and correction.

For robust image matching, outlier removal is essential. Methods such as RANSAC (RANdom SAmple Consensus) [16], and more recently MAGSAC [17] and BANSAC [18], are established practices that provide a method to discriminate against erroneous correspondences based on a model fitting process. However, the performance of these estimators can be compromised in certain cases. In scenarios with highly reflective surfaces, these algorithms may result in correspondences of high ambiguity; reflections can create false feature correspondences that mimic true matches, leading these algorithms to potentially erroneous conclusions. In addition to this, scenes with repetitive patterns are challenging due to the inherent difficulty in achieving a reliable geometric consensus. The patterns can cause propagation of feature matches that are statistically plausible yet geometrically incorrect, resulting in a consensus that does not accurately reflect the true alignment of images. Furthermore, the RANSAC-based methods’ assumption of uniformly distributed outliers does not hold in the presence of structured noise, a case common in industrial environments.

To this end, we categorize outliers as either background points or points on the vehicle surface. Point correspondences located in the background of the vehicle are separated from foreground features (on the vehicle) and excluded by leveraging two state-of-the-art learning-based frameworks, Yolo [19] and SAM [20]. Besides the background points, our approach introduces a novel filtering scheme that discards points extracted from the vehicle’s glossy surface, as they deviate from the dominant parallax vector (both magnitude and direction), to eliminate false matches by taking advantage of the constraints related to the motion of the vehicles. This approach plays a crucial role during image pose estimation towards creating the image mosaic.

Afterward, our approach stitches consecutive overlapping images to create a single high-resolution, wide-view image of the vehicle. Image stitching is a fundamental technique in digital image processing and has gained significant traction in various fields like remote sensing, virtual reality and medical imaging. A typical issue during the stitching process is the color differences between the stitched images. While traditional methods like pixel weighting have been employed for seam removal, more advanced techniques such as optimal seam methods [21,22] and transformation domain approaches (e.g., Fourier and wavelet transformations) [23,24] have shown improved results in creating seamless and visually coherent images. Despite considerable advancements in image stitching, challenges remain, particularly in scenarios with complex image content, dynamic scenes and 3D motion modeling requirements. In our study it is essential to maintain the originality of the image content; therefore, we utilize a low-level multi-band blending method following [25] that effectively minimizes the color differences but does not affect the integrity of the image as it is mandatory to keep the original content for the inspection process.

This work introduces an end-to-end pipeline for image matching and stitching concerning the inspection of moving vehicles that exhibit highly reflective surfaces. This work contributes to the field of exterior vehicle inspection by providing a comprehensive approach that leverages a recent image matching algorithm while introducing an effective outlier removal methodology. The principal outcome of this study is the demonstration of a viable, more efficient alternative to traditional vehicle inspection methods, which holds potential for widespread application in various sectors beyond the automotive industry. The findings of this research are expected to resonate with a broad audience, including those outside the immediate field of computer vision, due to their implications in enhancing safety, reliability and operational efficiency in exterior vehicle inspections.

The rest of the paper is organized as follows: In Section 2, we overview the related work and state our contributions briefly. In Section 3, we present the datasets that were used for experimentation. In Section 4, we present the proposed methodology for efficient image stitching. Section 5 presents the experimental setup and results. Section 6 briefly concludes the paper.

## 2. Related Work and Background

### 2.1. Vehicle Exterior Inspection

Vehicle exterior inspection plays a crucial role in maintaining road safety and validating the integrity of vehicles [26]. Traditional methods of manual inspection often involve time-intensive processes, high costs and a higher probability of human error. This not only delays and challenges the process but also raises questions about the precision and reliability of the inspection performed. As a result, there is an increasing demand for more efficient, accurate and automated approaches to vehicle inspection, such as in the context of insurance claims processing where rapid and accurate damage assessment is critical [27].

As an early approach, Ref. [28] delves into the need for effective vehicle undercarriage inspections through image mosaicking, especially in sensitive and high-security environments such as border control. In a more recent work [29], the focus shifts to enhancing security measures in restricted areas such as airports and embassies. This study integrates several computer vision applications, including license plate recognition, vehicle manufacturer/model detection and image mosaicking, to create a multi-faceted security system. Such systems are crucial for preventing fraudulent activities and ensuring that only authorized vehicles gain access to high-security zones. Other studies focus more on the insurance claims and road safety sectors. The authors of [30] present an innovative stereo-vision hardware solution for tire inspection through depth estimation, also utilizing deep learning models that include convolutional and recurrent network components. Their study highlights the importance of maintaining vehicle safety standards, showcasing how advanced technology can aid in the accurate identification of tire conditions, thus contributing to overall vehicle safety. Similarly, Ref. [31] copes with challenges in the insurance sector, particularly in assessing vehicle damage for claims processing, illustrating the potential for AI and DL in automating and improving the accuracy of damage assessment, thereby addressing issues like claims leakage and fraud in the insurance industry. The authors of [32] focus on automating the damage assessment process in the auto finance industry. Their research study emphasizes the need for precise damage estimation in leased vehicles, highlighting the challenges in designing robust systems that can accurately localize and measure damages under various conditions.

However, vehicle inspections introduce unique challenges that existing methods struggle to address effectively, and this is primarily due to the increased presence of reflections on the vehicle body. Extracting valuable information from moving objects like vehicles, especially in dynamic environments replete with reflections and varying lighting conditions, remains a formidable challenge. These dynamic conditions often lead to inaccuracies in defect detection, primarily due to the lack of proper image orientation, resulting in numerous false positives and necessitating additional manual intervention. In this context, the importance of advanced image matching techniques becomes paramount.

### 2.2. Image Matching

Image matching, a cornerstone of computer vision, involves identifying correspondences between two or more images and traditionally encompasses feature detection, description and matching. Recent review studies [33,34] provide a comprehensive analysis of the methods and application topics. Image matching has been extensively researched, and numerous handcrafted methods exist, with the most popular ones being SIFT [10], SURF [11] and ORB [12]. Recently, deep learning allowed the exploitation of more sophisticated approaches leveraging neural network architectures such as convolutional neural networks (CNNs), transformers, graph neural networks (GNNs) and very recently diffusion-based ones to build more resilient learnable solutions for image matching [12].

SuperPoint [13], as a pioneering work, introduced a self-supervised method for interest point detection by employing a fully convolutional neural network trained on a synthetic dataset and further refined through Homographic Adaptation. Moreover, the integration of this approach with a descriptor sub-network to attach fixed dimensional descriptor vectors to each point facilitates high-level semantic tasks such as image matching. This method significantly enhanced the performance of interest point detectors on real images as showcased by the authors.

The recently proposed vision transformer [35] is an extension of the original concept of transformers, used initially for natural language processing. Transformers introduced cascades of multi-head self-attention to compute representations of the input and output data without using RNNs or convolutions, currently being very popular for many computer vision tasks. LoFTR [14] presents a novel approach that increases efficiency compared to traditional local feature matching, which often fails in conditions like poor texture or repetitive patterns. LoFTR is a detector-free approach for feature matching that employs a CNN for early feature extraction and a transformer to identify semi-dense correspondences between two images. This method provides a substantial receptive field in the feature extraction network, which is crucial for distinguishing indistinctive regions, and shows effectiveness in challenging indoor and outdoor environments.

A more recent approach, TopicFM [36], introduced a novel perspective by combining local context and high-level semantic information into latent features for robust and accurate feature matching. Using a topic modeling strategy, the TopicFM method models images as a distribution over latent semantic instances, like objects or structural shapes. This probabilistic feature matching, based on the distribution of latent topics, enhances the distinctiveness of local visual features and achieves accurate dense correspondences, particularly in demanding scenes with large scale and viewpoint variations. RoMa [15], on the other hand, models image matching as a non-stationary diffusion process using a Markov chain formulation. Within this framework, two distinct stages emerge: the coarse matching stage, which prioritizes global consistency over precise localization due to the complexities posed by motion boundaries and repeated structures, and the refinement stage, which focuses on accurate localization once the initial coarse match is approximately correct.

Additional approaches focus on matching pre-existing features. SuperGlue [37], instead of focusing on improving local features and then applying standard matching heuristics, proposes learning the matching process using pre-existing local features (i.e., SuperPoint) through a novel neural architecture. It performs context aggregation, matching and filtering in a single end-to-end GNN architecture with self-attention (intra-image) and cross-attention (inter-image) inspired by the vision transformer for feature matching. LightGlue [38] extends this paradigm, addressing the computational intensity of deep matchers like SuperGlue. LightGlue is designed to be more accurate, more efficient and easier to train. Following an adaptive approach, it varies the computational depth based on the difficulty of each image pair, thus resembling human visual processing. LightGlue optimizes the trade-off between speed and accuracy, making it particularly suitable for real-time applications such as SLAM. RoMa outperforms all previous methods, demonstrating its superiority in the Image Matching Challenge [39]. Although RoMa produces adequate matches between two images, the extraction of features from moving objects with strong reflections introduces significant issues that existing image matching algorithms cannot tackle. Yet, most current methods assume a static environment, a presumption that rarely holds in real-world scenarios marked by constant motion and illumination changes.

### 2.3. Outlier Detection

Outlier detection among matched points is a decisive step in image matching and mosaicking since a single outlier might lead to incorrect adjustments. To tackle this, robust estimators like RANSAC [16] have become the gold standard. Since then, several approaches have been proposed to replace or extend the uniform sampling in RANSAC and increase the probability of finding an all-inlier sample and a good model in fewer iterations.

Robust estimators are categorized into heuristic-based strategies NAPSAC [40], PROSAC [41], and GroupSAC [42]; probabilistic-based strategies MLESAC [43] and EVSAC [44]; and learning-based strategies like NG-RANSAC [45] and NeFSAC [46].

MAGSAC++ uses a scoring function and a marginalization procedure. It does not require a single inlier–outlier threshold and can handle both noise and outliers effectively. It also introduces a new sampler, the Progressive NAPSAC, that exploits the spatial coherence of the data and transitions from local to global sampling. Graph-Cut RANSAC [47] utilizes the graph-cut algorithm to separate inliers and outliers in a local optimization step, which is applied when a so-far-the-best model is found. As a recent improvement in this area, BANSAC [18] employs a dynamic Bayesian network for adaptive sample consensus iteratively updating the inlier probabilities of data points during the RANSAC iterations. This adaptive mechanism augments the accuracy of feature matching while enhancing computational efficiency. In our approach, we utilize MAGSAC++ to perform geometric verification of the extracted features. Robust estimators are essential for proper model fitting and geometric verification; however, the repetitive patterns and reflections still may lead to inaccurate geometric rectification. To address this, we initially extract the background of the scene to eliminate points from the vehicle’s background, and additionally, we introduce a novel filtering module that includes an ensemble of statistical methods to remove the points on the glossy surface of the vehicle’s body.

Applying statistical-based outlier detection methods such as those in [48,49] is a common practice that improves model fitting. Additionally, many ensemble strategies employ combinations of these methods for increased robustness and adaptiveness [50,51]. In-depth studies [52,53,54,55,56] demonstrate the importance of removing statistical outliers for a variety of tasks. In most cases, it is essential to select and primarily use specific filtering methods that are appropriately suited to the nature of the data. The authors of [57] categorize outliers as (a) single-point outliers, (b) collective outliers and (c) contextual outliers. The single-point outlier is an individual data instance that deviates from the rest of the dataset, collective outliers are a collection of data instances that appear anomalous with respect to the rest of the entire dataset and contextual outliers are anomalous data instances in a specific context or neighborhood.

In image matching, there are many approaches that utilize statistically based outlier detection methods. The authors of [58] introduce the Guided Local Outlier Factor (GLOF), an adaptation of the Local Outlier Factor (LOF), a density-based scoring mechanism commonly used in anomaly detection within data mining. The GLOF modifies the LOF for feature matching by embedding feature vectors from x to y into a 2D spatial distribution. This enables the calculation of a score for each match, indicating its validity as true or false. Another approach [59] reduces redundant keypoints using principal component analysis (PCA) to downscale the DBSCAN clustered features. The authors of [60] propose the use of an iterative spatial clustering approach assuming that there exists consistent motion among the potential matches in an image pair.

Feature matches can fall into these three categories as they inherently possess both spatial properties from the image and can be affected by noise. For example, a particular sub-group of matches can be considered as collective outliers if they exhibit behavior that is distinct from the rest of the data due to motion patterns. Moreover, image matches can be identified as contextual outliers when their appearance is specific to a particular context within the image, e.g., a glossy surface. Finally, single-point outliers can be distinguishable points possibly due to noise, detection errors, hot spots and so on. Ultimately, these outliers can be effectively analyzed using parallax vector analysis, as the motion between the images is predominately planar and characterized by a dominant motion vector.

The characteristics of the current data have guided the design of our ensemble approach, which combines three density-based clustering methods and three statistical-based methods. This effectively removes outliers that are spatially correlated, such as those on glossy surfaces or deflectometry stripes, as well as isolated single-point outliers.

### 2.4. Image Stitching

Image stitching plays a critical role in a diverse array of applications ranging from autonomous vehicle driving and navigation [61,62] to augmented reality [63], medical imaging [64,65] and vehicle exterior monitoring [29]. Traditionally, image stitching involves aligning and merging multiple images taken from different perspectives of the same point into a seamless, high-resolution panorama or mosaic. However, in many cases, the motion of the camera is not only rotary but also includes a translation.

One of the main challenges in image stitching is ensuring a natural and seamless blend of images, particularly in the presence of parallax, lens distortions and varying scene illuminations/reflections. Traditional methods typically rely on global 2D transformations and thus must cope with misalignments and ghosting effects, where duplicate objects appear in the mosaic. The complexity of accurately estimating the stitching field, influenced by the intricate interplay between the 3D scene structure and camera parameters, adds to these challenges.

Recent advancements in deep learning have significantly improved image stitching techniques [66] to enhance feature extraction, homography estimation and image blending. These models are adept at handling changes in noise, lighting and occlusions, making the stitched images more realistic and robust against common stitching artifacts.

## 3. Datasets

**Figure 1:**
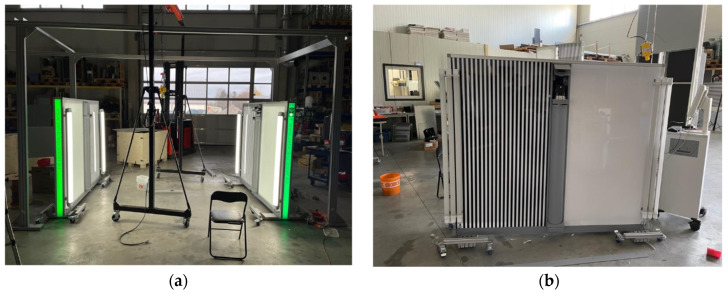
(**a**) Experimental Two Tronic’s scanner; (**b**) master side of the Two Tronic scanner.

The datasets explored in this study consist of images captured in a real-life scenario where driver behavior was not strictly predefined, and image acquisition characteristics were completely unknown. Occasional blurring of images, along with reflective stripes on the vehicles, disrupts the feature extraction and matching process. The relatively featureless large uniform surfaces of vehicles further complicate the matching task. All image sequences were collected using an industry-ready scanner as shown in Figure 1, which includes a LIDAR line sensor for triggering purposes, LED illumination and deflectometry stripes [67].

Image Set: *Volkswagen*

The *Volkswagen* dataset consists of 16 images of 3000 by 4096 pixels and presents the left side of a vehicle. The images were collected in an outdoor environment.

**Figure 2:**
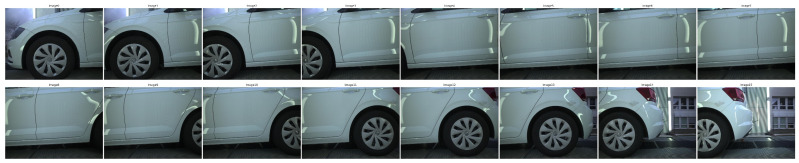
Image dataset “Volkswagen”.

**Figure 3:**
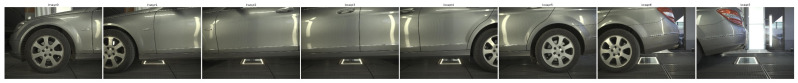
Image dataset “Mercedes”.

**Figure 4:**
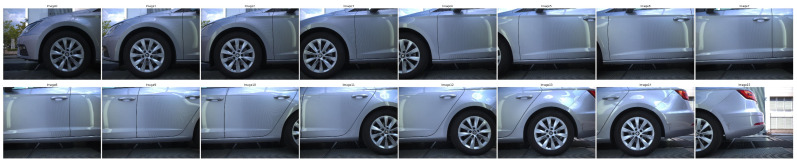
Image dataset “Seat”.

Experiments were conducted on three image sequences: *Volkswagen* (Figure 2), *Mercedes* (Figure 3) and *Seat* (Figure 4). Each dataset presents unique challenges, primarily due to variations in image overlap, quality and indoor/outdoor environmental conditions. For instance, the *Mercedes* dataset maintains less than 65% overlap between consecutive images, while the other two maintain more than 80%. Another significant difference between the *Mercedes* and the *Volkswagen* and *Seat* image sets is that the latter two were collected in an outdoor environment leading to different lighting conditions. For all three datasets, the cameras were fixed while the vehicle was moving.

Image Set: *Mercedes*

The *Mercedes* dataset consists of 8 images of 3000 by 4096 pixels and presents the left side of a vehicle. The images were collected in an indoor environment.

Image Set: *Seat*

The *Seat* dataset consists of 16 images of 3000 by 4096 pixels presenting the left side of a vehicle. The images were collected in an outdoor environment.

As already mentioned, one of the main issues for all image datasets is the poor textural variation of vehicle surfaces. Another significant characteristic is the absence of camera calibration data and control points, typically used for image orientation and scale recovery. All vehicles were moving along the *x*-axis while the camera remained fixed. The images display significant variations in lighting and reflective patterns.

## 4. Methodology

**Figure 5:**
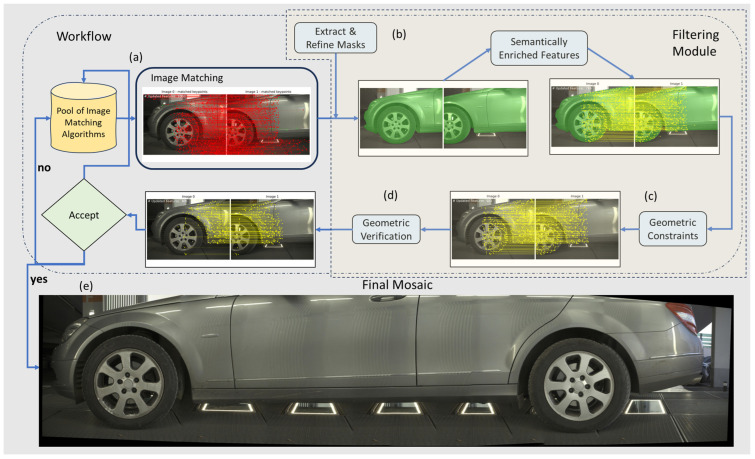
This diagram illustrates the workflow of our approach. (**a**) Image matching is performed using one algorithm from the pool of image matching algorithms (initially, RoMa). (**b**) The next phase involves extracting and refining masks that filter out the points in the background. (**c**) The remaining features are subjected to the filtering module, where geometric constraints are applied. (**d**) Following the filtering module, a geometric verification is conducted to ensure the geometric accuracy of the result. (**e**) The outcome of this process is a final, seamless mosaic.

Given a set of high-resolution images of vehicle exteriors, our aim is to develop an automated process for image matching and stitching to increase the effectiveness of vehicle inspection, considering the unique lighting conditions. Our work focuses on seamlessly stitching images to create a cohesive panoramic view of the vehicle. In the proposed framework (Figure 5), we integrate three dense feature matching methods in the “Pool of Image Matching Algorithms”, i.e., RoMa, TopicFM and LoFTR. This can be leveraged in case RoMa underperforms due to unspecified issues leading to a false geometric verification. For each consecutive image pair, we extract and refine the vehicle’s masks to eliminate points located on the background of both images. Subsequently, we filter out matches based on the dominant parallax vector (based on the calculated relative translation t of the image pair). The remaining matches are then geometrically verified. The acceptance or rejection criterion of an oriented image pair is the magnitude of the Euler angles, derived from the rotation matrix **R**, and of the translation vector **t**, extracted by decomposing the essential matrix **E** of the particular image pair. The final mosaic is produced by sequentially stitching consecutive pairs using their median parallax, which is the median translation distance between the final matches in each image pair.

### 4.1. Image Matching

Prior to choosing RoMa as the main dense feature matcher of our framework, we conducted several experiments between four state-of-the-art image matching algorithms, i.e., RoMa, LoFTR, TopicFM and SuperPoint + LightGlue.

To evaluate these algorithms, we used their respective GitHub implementations [68,69,70,71] without any fine-tuning.

**Figure 6:**
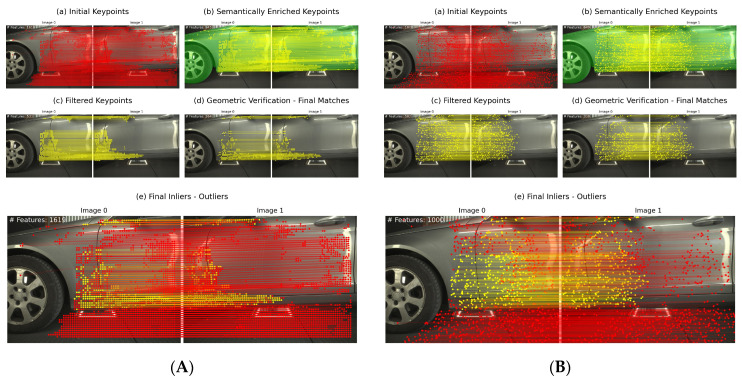
(**A**) Proposed method utilizing TopicFM; (**B**) proposed method utilizing RoMa.

**Figure 7:**
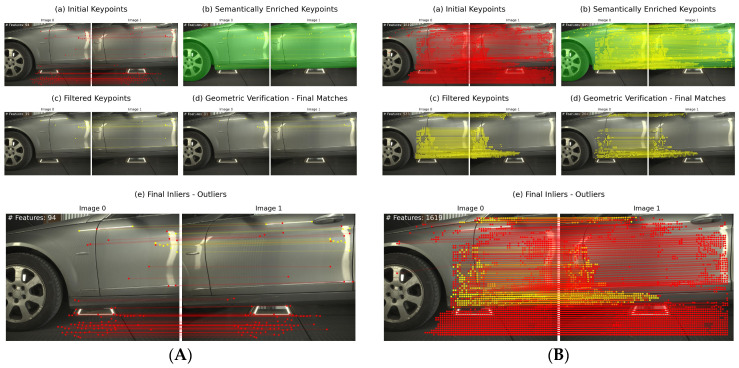
(**A**) Proposed method utilizing SuperPoint + LightGlue; (**B**) proposed method utilizing LoFTR.

All methods extract features on the background, which need to be removed for reliable image pair orientation. Figure 6 and Figure 7 illustrate the results of our method employing TopicFM against RoMa and SuperPoint + LightGlue against LoFTR, respectively. In our experiments, dense matchers surpassed SuperPoint + LightGlue as the latter provided too few matches. It is observed that TopicFM and LoFTR both tend to detect keypoints following a grid pattern. Dense matchers generally yield a substantial number of features; however, post-filtering is essential for ensuring a higher accuracy in the matching process.

After initial keypoint extraction, we eliminate background points from each set using the extracted masks (see Section 4.2.1). Next, we apply the proposed novel filtering scheme (see Section 4.2) to discard points that deviate significantly from the dominant parallax vector (checking both magnitude and direction) to remove false matches, such as points matched on the opposite sides of the images or points matched diagonally. In this way, we exploit the vehicle’s motion as a critical geometrical prior. Finally, we utilize MAGSAC++ for robust fitting in estimating the essential matrix, thereby accurately recovering the relative orientation of each image pair.

### 4.2. Filtering Module

We propose a novel filtering module for use in image matching. This module is applicable when the number of features is adequate. Intuitively, if only a few matches are extracted, e.g., ~30, the filtering scheme may underperform due to the poor distribution of points in the overlapping area of the image pair (which may well include ambiguous matches due to glossy surfaces and repetitive patterns). In this step, feature points on the background of the scene are separated from foreground features (on the vehicle) and excluded. Besides the background points, the algorithm also discards points extracted from the vehicle’s glossy surface, as they deviate from the dominant motion.

Additionally, as mentioned, we exploit the dominant parallax vector (both magnitude and direction) to eliminate false matches, taking advantage of the constraints related to the motion of the vehicles. Overall, we introduce the following distinct outlier removal processes:

- Background points: We apply semantic segmentation and object detection using two state-of-the-art learning-based frameworks, YOLOv8 and SAM.
- Points on highly reflective areas: We detect points with very small or extreme parallax. Points with zero or relatively small parallax, apart from those of the static background, also refer to scene points that have been reflected on the glossy surface of the vehicle. These points, rather than following the vehicle motion, maintain the same or nearly the same position across frames, with only minor variations due to the vehicle’s surface differentiation.
- Points with random parallax: we introduce a filtering scheme that estimates a dominant parallax vector, primarily aligned with the image *x*-axis. This is a key component in the outlier removal step and is particularly crucial in contexts where understanding the orientation of features relative to a reference axis is essential. Our main focus is on identifying and retaining those features that closely align with a specific motion vector (i.e., *x*-axis) and magnitude of the dominant parallax vector. By setting a threshold (2 degrees) for angular deviation from the dominant parallax vector and applying the proposed filtering scheme, we filter out points that do not conform to the desired horizontal alignment or the common vehicle motion. Due to our system’s configuration, vehicles are moving along the *x*-axis. Nonetheless, our filtering scheme is adaptable to various camera setups, not limited to those strictly aligned with the *x*-axis, provided that a dominant motion vector is present.

#### 4.2.1. Semantically Enriched Features

Our approach enriches the matches with semantic information as it integrates two segmentation processes using neural networks and post-processing techniques.

YOLO is a popular deep learning model used for object detection, capable of identifying and classifying various objects within an image in a single forward pass. The architecture is composed of a series of convolutional layers that extract features from the image, which are then used to predict bounding boxes and corresponding class probabilities.

Segment Anything Model (SAM) utilizes a prompt-based architecture designed for customizable segmentation tasks without the need for retraining on new datasets. SAM excels in generating high-fidelity masks for a variety of objects within images, even when object categories are provided during inference via textual descriptions. This prompt-based method is flexible and efficient, allowing SAM to segment new object classes not encountered during its training phase. SAM implements a vision transformer (ViT) as its backbone. Vision transformers have gained prominence in the field due to their self-attention mechanisms, which enable them to focus on different parts of the image to extract rich contextual information that improves segmentation performance. Unlike convolutional networks that process the image in a somewhat local manner, transformers perceive the image globally through self- and cross-attention, which is particularly advantageous for segmentation tasks where understanding the entire context of the image is important.

**Figure 8:**
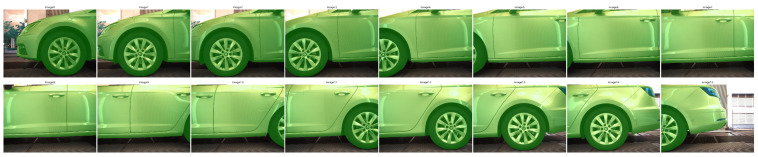
Extracted masks for the *Volkswagen* dataset. We first extract masks using Yolov8, and then we refine these using SAM.

In our integrated pipeline, YOLOv8 identifies objects and their corresponding segmentation masks. Then, these masks are prompted to SAM, with the latter providing a fine-grained segmentation of the detected objects (Figure 8). SAM operates by producing embeddings for the regions of interest prompted by the YOLOv8 detections. It then generates detailed masks by classifying each pixel within the region, resulting in precise segmentation even in complex visual scenes.

The process involves converting SAM into the ONNX format, which is a cross-platform model representation that enables efficient execution on various hardware accelerators. Moreover, the model undergoes dynamic quantization, which improves computational efficiency without a significant loss of accuracy. This optimization is essential for deployment in resource-constrained environments or real-time applications.

In summary, our methodology combines the real-time detection capabilities of YOLOv8 with the flexible, high-fidelity segmentation provided by SAM. Together, they form a robust system for analyzing and interpreting visual data.

#### 4.2.2. Outlier Detection

**Figure 9:**
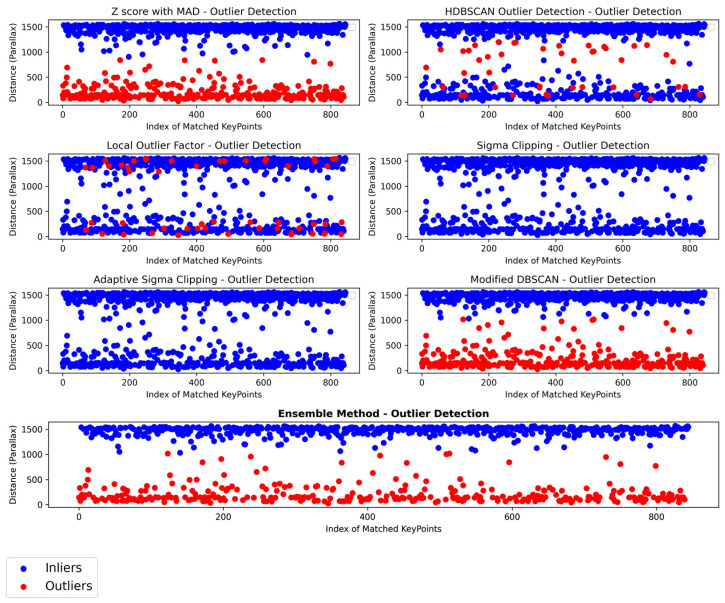
Ensemble for outlier detection: **Top left**: Z-score with median absolute deviation (MAD) identifies outliers based on standardized deviation from the median. **Top right**: HDBSCAN (Hierarchical Density-Based Spatial Clustering of Applications with Noise) detects outliers through hierarchical clustering. **Middle left**: Local Outlier Factor determines outliers by measuring the local deviation of a given data point with respect to its neighbors. **Middle right**: Sigma clipping uses standard deviation thresholds to identify and remove outliers. **Bottom left**: Adaptive sigma clipping dynamically adjusts sigma thresholds based on the distribution of distances. **Bottom right**: Modified DBSCAN (Density-Based Spatial Clustering of Applications with Noise) employs a density-based approach leveraging the median value. The ensemble method (**bottom center**) combines the results of individual methods to achieve a consensus-based outlier detection. A match is an outlier if more than two methods of the ensemble mark it as an outlier.

The proposed ensemble for outlier detection (Figure 9) includes six different statistical methods, i.e., two clustering methods, modified DBSCAN and HDBSCAN; Z-score with median absolute deviation (MAD), sigma clipping, and adaptive sigma clipping, which are simple yet effective ways to remove outliers based on the median value; and Local Outlier Factor (LOF).

The Z-score with MAD is robust against outliers, making it particularly suitable for datasets with a few extreme values. On the other hand, it assumes a unimodal distribution centered around the median, which can be a limitation, and thus may fail in multimodal distributions or when outliers do not significantly deviate from the median, especially in datasets with multiple clusters where the median is not a meaningful measure.

The Local Outlier Factor (LOF) is effective in datasets where local density varies, and it is adept at detecting outliers that are anomalous in their local neighborhood. Nonetheless, the LOF is sensitive to the choice of parameters, like the number of neighbors, and may struggle in high-dimensional spaces or when datasets preserve a uniform density where distance metrics become less meaningful.

Sigma clipping is simple yet effective for symmetric, unimodal distributions. It operates under the assumption that the majority of data points are not outliers, which might not always hold true. Hence, it is less effective in heavily skewed datasets or in datasets where a significant proportion of the data are outliers. Similar to sigma clipping but with a dynamically adjusted threshold, adaptive sigma clipping offers more flexibility compared to standard sigma clipping, due to its dynamically adjusted threshold that adapts to the data. Despite its adaptability, it can still face problems with multimodal distributions or very noisy datasets, and this method may fail in datasets lacking a clear ‘central’ cluster or where the noise level is too high for meaningful adaptation, leading to many false outliers.

In this work, we propose the modification of DBSCAN to use the median as a baseline for point clustering, which is particularly good for spatial data and datasets with clusters of varying density. It is sensitive to parameter settings, and it might fail when applied to datasets with highly irregular densities.

To address this, we include HDBSCAN, which improves upon DBSCAN by handling varying density clusters more effectively. The method can be computationally intensive and might yield varying results with different data scales, and it may not perform well in datasets where cluster boundaries are not well defined or in extremely large and complex datasets where computational resources are a constraint. We utilize all the aforementioned methods in an ensemble to increase the confidence of our framework for outlier detection. The chosen methods complement each other in handling different aspects and types of outliers.

##### Ensemble Method

Our ensemble method incorporates six distinct methods. For single-point outliers often resulting from noise, detection errors or unique image features, we employ Z-score with median absolute deviation (MAD), sigma clipping and adaptive sigma clipping. These approaches are efficient in detecting deviations from a baseline, which normally is the median value of the dataset. The LOF extends the ensemble by assessing the local density deviation of a point with respect to its neighbors, thus enabling the detection of both single and contextual outliers. Finally, our ensemble includes modified DBSCAN and HDBSCAN versions that can identify both collective and contextual outliers.

Consider the ensemble E consisting of N outlier detection methods:

(1) $$E=\left\{M_{1},M_{2},\ldots ,M_{N}\right\}$$

For a given parallax vector x, let the vote of method $M_{i}$ be denoted as $V_{M_{i}}\left(x\right),$ where

$V_{M_{i}}\left(x\right)=1$ if $M_{i}$ classifies x as an inlier and $V_{M_{i}}\left(x\right)=0$ if $M_{i}$ classifies x as an outlier. The ensemble vote E(x) is then defined as

(2) $$E\left(x\right)=\{\begin{array}{c} 1\\ 0 \end{array}\begin{array}{c} \;\;\;\;if\;\sum_{i=1}^{N}V_{M_{i}\left(x\right)}\;\geq \;T\\ otherwise \end{array}$$

where T is the minimum number of methods that must agree on classifying the parallax vector of each point pair as an inlier.

**Figure 10:**
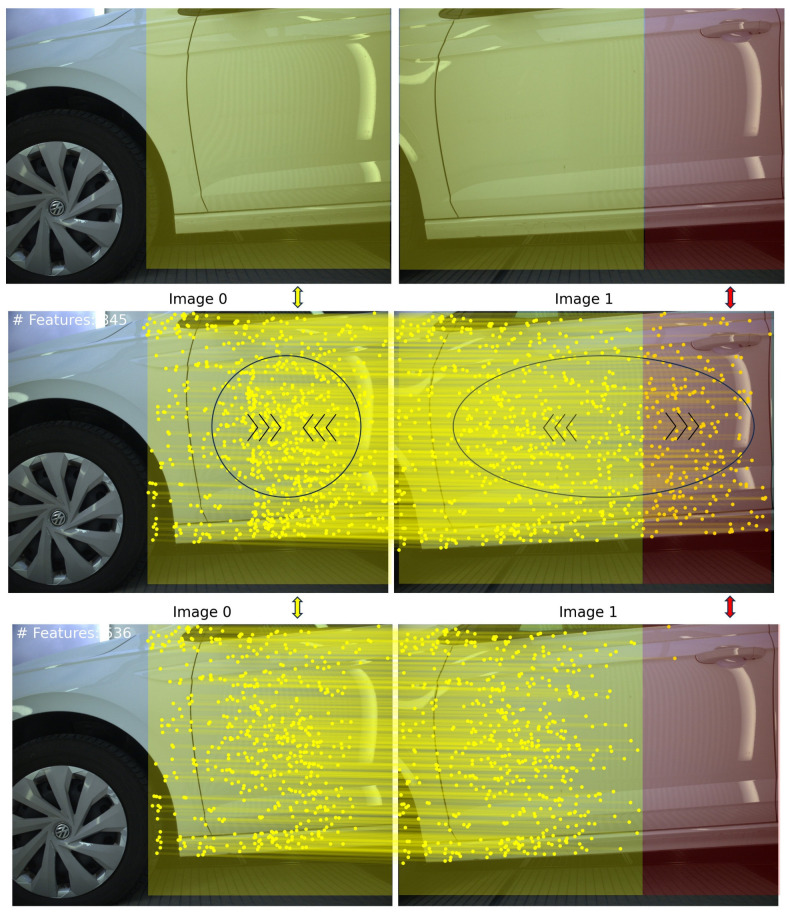
**Upper** Image Pair: Yellow transparent areas show the overlapping area while red transparent area shows the non-overlapping area. **Middle** Image Pair: Arrows emphasize the density of the matches initially proposed by RoMa. Points are being squeezed in the left image, while those in the right image are dispersed. **Bottom** Image Pair: The matches after our filtering module is applied.

**Figure 11:**
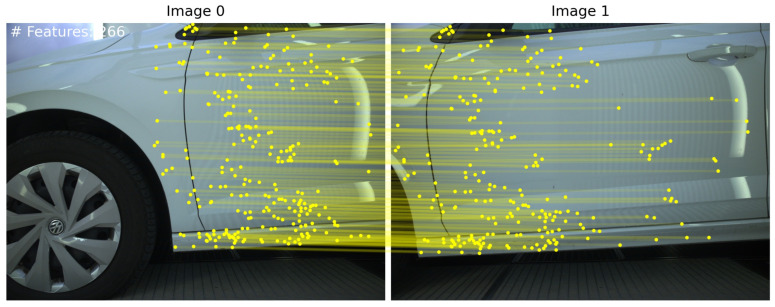
Skipping filtering results in false geometric verification.

Our ensemble integrates multiple statistical outlier detection methods to benefit from their collective strengths, resulting in a robust, aggregated method for identifying outliers as shown in Figure 10. We note that this method is efficient when a specific dominant parallax vector is present such as in our case. This filtering process is critical, and skipping it may lead to erroneous results as shown in Figure 11.

#### 4.2.3. Geometry Verification

After applying the ensemble to the parallax vectors for outlier removal, geometric verification is mandatory.

The initial step involves applying MAGSAC++ for outlier rejection; to do so, we rely on OpenCV’s USAC framework [72]. Unlike conventional RANSAC, MAGSAC++ adapts the inlier–outlier threshold dynamically, making it particularly effective in scenarios with a significant outlier ratio. MAGSAC++ implements a marginalization procedure over a range of possible thresholds to determine the most probable inlier set, thus optimizing the selection process.

**Figure 12:**
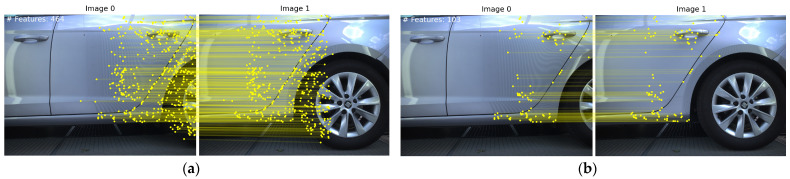
(**a**) Keypoints filtered using our proposed method. (**b**) Final matches after geometric verification.

Upon obtaining a purified set of inliers from MAGSAC++ as shown in Figure 12, we proceed to estimate the essential matrix of image pairs using the LMEDS approach. LMEDS is chosen for its robustness in handling datasets with the presence of remaining outliers and works by finding the median of the squared residuals. This method is less sensitive to outliers compared to traditional least squares, making it well suited for scenarios where outlier contamination, although reduced, is still a concern. Camera calibration is unknown. In order to estimate a common global focal length, we employ the well-known approach of Bougnoux [73] and accordingly assume that the principal point coordinates for each camera and the distortion coefficients are zero.

The camera pose, i.e., the rotation matrix R and the translation vector t, is recovered from the essential matrix E. Following [74], we first decompose the essential matrix using singular value decomposition (SVD) to obtain four solutions (two rotation matrices and two translation vectors). The combination of R and t that satisfies the cheirality constraint, i.e., object points should be in front of both cameras, is selected. A pair is accepted or rejected depending on the Euler angles which are derived from R. If the absolute values of these three angles are below a threshold (in our experiments, it was set at 4 deg), the image pair is accepted.

## 5. Results

**Figure 13:**
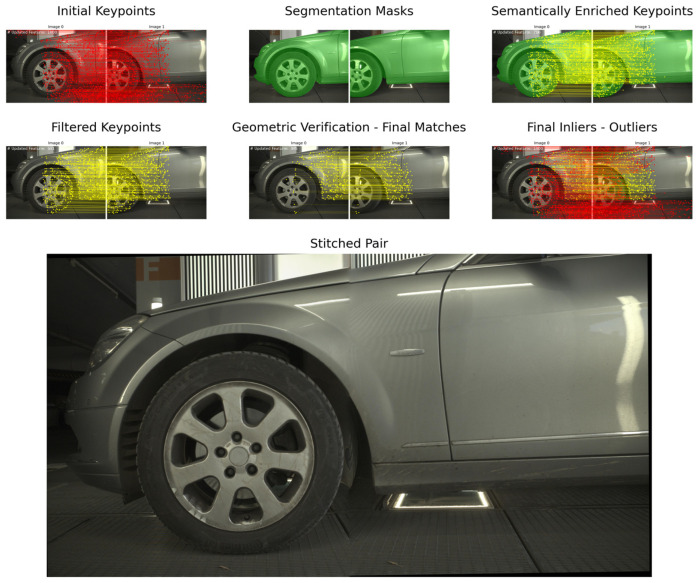
The proposed workflow for an image pair of the dataset *Mercedes*.

An example of our workflow for an image pair of the *Mercedes* dataset is presented in Figure 13. Despite the changes in illumination, RoMa locates adequate points for matching; however, applying our filtering scheme and geometric verification is essential. The extraction of segmentation masks allows the elimination of the points in the background, thus reducing the number of outliers. Dense matchers tend to produce a vast number of features, but in our experiments, we limited the number of extracted matches to 1000. The geometric verification process discards points on the wheels but does not fully eliminate them. The ratio between final inliers and outliers is significant, which clearly shows the difficulty of extracting correct matches under such environments. Finally, the multi-band blending approach limits the color differences among images. As all datasets include images of large size (3000 × 4096 pixels), the process is computationally costly. To tackle this, we re-implemented multi-band blending in pytorch to run entirely on a GPU.

**Figure 14:**
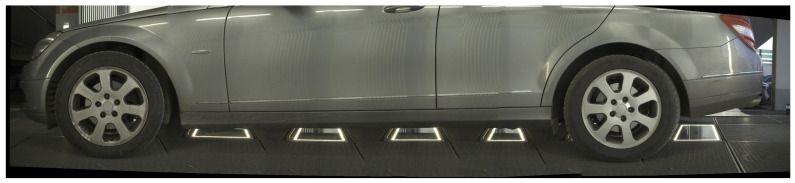

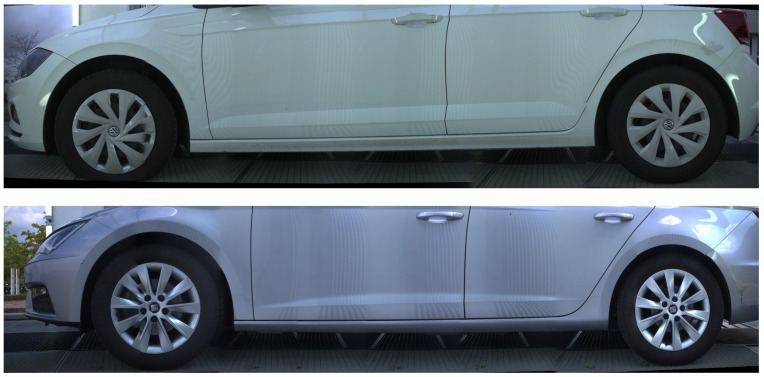
Upper: Image mosaic for the *Mercedes* dataset. Middle: Image mosaic for the *Volkswagen* dataset. Bottom: Image mosaic for the *Seat* dataset.

All image sets were matched, filtered and stitched fully automatically. All tasks, i.e., image matching, object detection, segmentation and filtering, along with the final image mosaics for the three datasets shown in Figure 14, were processed on a laptop with an i7-11800H and an Nvidia RTX 3060 GPU.

The movement of a vehicle’s wheel represents a separate challenge. Unlike the rest of the vehicle, which undergoes a single motion relative to the camera, the wheels exhibit a rotational movement. This creates discrepancies and artifacts in the stitched image, particularly noticeable at the seamlines. This issue is compounded by the fact that wheels often contain intricate patterns (such as spokes or hubcaps) that are prone to creating complex and misleading features for matching algorithms. As a result, the wheels might appear blurred, distorted or misaligned in the final stitched image, which can significantly impact the accuracy and reliability of the inspection process. Owing to this, all the points located on the wheel are considered as outliers and must be removed.

**Figure 15:**
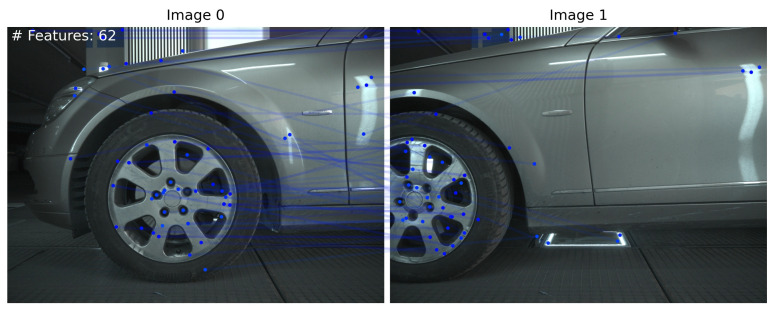
Image matching using SIFT algorithm.

Current tools for image stitching do not integrate recent image matching algorithms, nor do they employ specific statistical methods for outlier filtering based on motion patterns. As illustrated in Figure 15, SIFT fails to produce a sufficient number of features for effective image stitching. Thus, it is not possible to make a specific qualitative or quantitative comparison between our method and the existing tools.

## 6. Conclusions

Computer vision and deep learning have transformed vehicle exterior inspection processes, providing more reliable, faster and cost-efficient solutions. The automation of defect identification utilizing a seamless image mosaic could streamline the inspection process and increase the accuracy of damage assessment. Currently, such solutions grapple with persistent limitations due to reflective patterns, complex environments and motion presence, thus requiring manual refinements. The proposed framework could potentially be used for numerous possible applications in various sectors. For example, it can be applied within the transportation and logistics industry for fleet vehicle inspections. Additionally, its application extends to industrial environments for the inspection of shiny objects, e.g., metallic components, employing a similar camera setup.

In this work, we have proposed a novel image matching and stitching framework that builds upon RoMa, Yolo and SAM. A critical component of the framework is the introduced filtering ensemble. Applying such statistical-based outlier detection methods is an underexplored topic in image matching. The available image stitching approaches rely on handcrafted image matching algorithms, thus making the direct quantitative comparison with the proposed approach not possible. Additionally, to the best of our knowledge, there is no suitable dataset with available ground truth that would allow us to effectively evaluate our approach.

A more thorough evaluation could be based on either a synthetic [75,76] or a real ground truth dataset. For the latter, the goal would be to capture several synchronized 3D scans and image frames of a slowly moving vehicle. The geometrical calibration of the two sensors (laser scanner and camera) would allow the accurate positioning of every camera frame with respect to the vehicle coordinate system.

A potential future task could also be the combination of statistical and geometric filter detection methods within a neural network, e.g., a multi-head transformer architecture that clusters points based on motion patterns to filter out points that differentiate from their neighbors. Transformer architecture enables the model to weigh the importance of different features on an image in relation to each other through self-attention. Multi-head attention extends this by allowing the model to attend to different parts of the input in parallel. Incorporating a spatially aware filtering scheme to iteratively group matches based on their distinct motion pattern could potentially provide more robust insights to the network and might lead to faster convergence and more robust outlier removal as well.

The wheels of the vehicle follow a different rotary motion compared to the vehicle’s body. Addressing this challenge requires a specialized approach. A potential future task of our research could involve the development of a ‘detect and remove’ strategy, leveraging advanced image detection techniques. This approach would involve identifying wheels and then applying specialized algorithms or transformations to account for their rotational movement. To efficiently place the wheel on the panorama, a possible solution could be to initially determine the center of each detected wheel assuming the wheel has a circular shape. Then, based on the centers of all detected wheels, we could estimate the midpoint and place the wheel following the specific perspective transformation. By isolating these areas and treating them differently from the rest of the vehicle during the stitching process, we could potentially reduce or eliminate the artifacts caused by wheel rotation.

For a more accurate image mosaicking result, the bundle adjustment method can be applied for global image pose and camera calibration refinement. Bundle adjustment could optimize pose estimation and reduce the geometric ambiguities often associated with relative pair geometry. In this case, a current challenge lies in effectively matching keypoints extracted by dense matchers, as they typically do not involve feature descriptors. By employing bundle adjustment, we can create a finer, seamless and geometrically consistent image mosaic that can be particularly beneficial in scenarios where multiple images of a vehicle are taken from different viewpoints.

Finally, a neural implicit representation method (NeRFs) [77] can be implemented for a more holistic high-fidelity 3D representation of the vehicle. These methods surpass traditional 3D reconstruction techniques in realism and have recently become more efficient with sparse imagery, thus requiring less extensive viewpoint sampling.

## Data Availability

The dataset used for this article is strictly for this research material, and commercial exploitation is prohibited. All datasets are provided by TwoTronic GmbH and consist of real images collected for an actual vehicle inspection process.
